# Implications of Fagopyrin Formation In Vitro by UV Spectroscopic Analysis

**DOI:** 10.3390/molecules26072013

**Published:** 2021-04-01

**Authors:** Anatolij Kosyan, Oksana Sytar

**Affiliations:** 1 Department of Plant Biology, Educational and Scientific Center “Institute of Biology and Medicine”, Taras Shevchenko National University of Kyiv, Hlushkova Avenue, 2, 03127 Kyiv, Ukraine; a_kosyan@ukr.net; 2 Department of Plant Physiology, Slovak University of Agriculture in Nitra, A. Hlinku 2, 94976 Nitra, Slovakia

**Keywords:** naphthodianthrones, fagopyrin, hypericin, *Fagopyrum*, differential spectrophotometry

## Abstract

The present work aims at studying the possible biosynthesis of fagopyrin in buckwheat plants with an attempt to address the existing gaps. The developed method of differential spectrophotometry can be used for identification of naphthodianthrones fagopyrins. It was found that in the vegetative mass of buckwheat plants, fagopyrin precursor-2-(piperidine-2-yl)-emodindianthron could be present. As fagopyrin can be produced by light effect, the temperature factor may influence the formation of protofagopyrin in vitro. An optimum temperature range was estimated for protofagopyrin formation. A possible fagopyrin biosynthesis under in vitro conditions was suggested.

## 1. Introduction

Buckwheat (*Fagopyrum esculentum* L.), a traditional pseudocereal crop (*Polygonaceae*), is widely known as a food and medicinal plant. Different parts of Buckwheat plants, especially inflorescences, have already been studied for flavonoids and phenolic acids [1,2]. Buckwheat is expected to be an even more important plant in agriculture as a food crop as it is part of the dietary habits in many countries, including those of Europe, Asia and America [3]. Buckwheat research has gained significant interest recently due to the presence of some less studied secondary metabolites with antioxidant and anticancerogenic effects [4,5,6].

Fagopyrin is a naphthodianthrone with anticancerogenic effect, which was isolated for the first time in 1943 from the blossoms of the red flowering variety of *Fagopyrum esculentum*, a plant known since 1833. The chemical structure of fagopyrin (red, fluorescent pigment) has been deduced only in 1979 [7]. The structure of fagopyrin is very similar to that of hypericin, which is present in St. John’s wort (*Hypericum perforatum* L.). The aromatic structure of fagopyrin is like that of hypericin, differing only in the presence of two symmetrically placed 2-piperidinyl groups in fagopyrin.

Naphthodianthrone compound present in Fagopyrum sp. (fagopyrin, fagopyrin derivatives) have a photosensitizing effect [8] which is currently used for photodynamic therapy of cancer cells [9]. fagopyrin and hypericin have also shown antifungal effect on pathogenic fungi and spoilage yeasts [10,11]. Recent studies have identified fagopyrin by HPLC [12,13] but its exact quantification, isolation and biosynthesis is still under discussion. Tavčar Benković et al. (2014) with the help of HPLC analysis have determined the structures of two new derivatives (fagopyrin A and fagopyrin E) and proved the existence of protofagopyrin that can transform into fagopyrins upon light exposure [14]. Therefore, in the present work, we have attempted to study ways of fagopyrin formation with the aim to address the gaps in fagopyrin biosynthesis.

The highest quantity of fagopyrin derivatives was observed in inflorescences and lower amount in leaves of common buckwheat [15]. Ozbolt et al. (2008) found in leaves of common buckwheat (*Fagopyrum esculentum* L.) 3 times higher fagopyrin content compared to the stems [16].

The development of an effective method to determine the quantity of fagopyrin was problematic as the buckwheat leaf extracts contain a considerable amount of chlorophyll [17]. The content of fagopyrin derivatives can be determined with UV–vis spectrometer at 590 nm, i.e., the wavelength used for evaluating hypericin concentrations [16,18]. Differential extraction of flavonoids and fagopyrin derivatives from the green parts of buckwheat is possible by the adjustment of extraction conditions [18]. Until recently, the isolation and extraction attempts of fagopyrin was under development with the use of spectrophotometric, TLC and HPLC analysis [14]. The preliminary analysis of previous studies with known parameters of extraction of fagopyrin for spectrophotometric analysis is shown in Table 1. As we see in the study of Sha et al., 2018, fagopyrins in buckwheat leaves was extracted by 90% acetone, methanol, 60% glacial acetic acid and 80% tetrahydrofuran. Then, 90% acetone was used as the best extractant, and UV–vis spectrophotometry was considered the simple and fast analytical method [19].

This paper focuses on designing a development method of differential spectrometry analysis to examine naphthodianthrones in buckwheat plant extract with 90% aqueous acetone. This method consists of a combination of differential spectrometry that takes into account the effects of temperature and light. The possible steps of fagopyrin biosynthesis in intact plants and under in vitro conditions, which can be affected by temperature and light, were described.

## 2. Results

**Figure 1:**
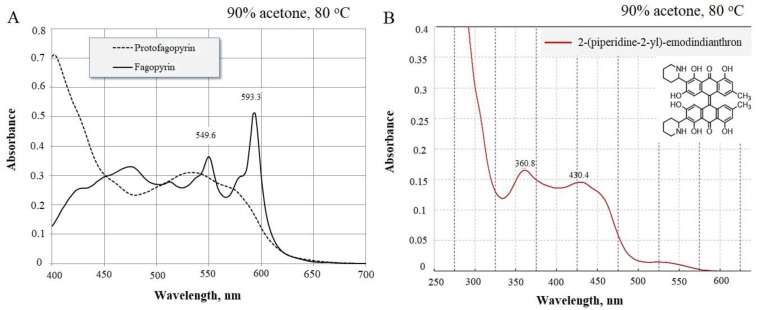
Absorbance spectrum of identified protofagopyrin and fagopyrin spectrum in vitro (**A**) and 2-(piperidine-2-yl)-emodindianthron in intact plants (**B**) (method of differential spectrophotometry).

Quantitative analyses often involve the spectrophotometric resolution of mixtures of two components with partly overlapping spectra, and the greater the extent of overlap, the more difficult the resolution. Not surprisingly, this topic has been the subject of a number of chemometric studies originally intended for the resolution of binary mixtures and later extended to three or more components [20,22,23]. The use of differential spectrometry for the quantitative determination of the presence of napthdiantrone fagopyrin and its precursors showed that in intact buckwheat plants extracts, the 2-(piperidine-2-yl)-emodindianthron-(Z)-2,24,4’,5.5’-hexahzdroxy-7,7’-dimetyl-3,3’-di(piperidin-2-yl)-10H,10’H-[9,9’-bianthacenylidene]-10,10’-dione (Figure 1) was mostly present.

The spectrum for the identification of fagopyrins group was in the range from 360.8 nm to 430.4 nm (Figure 1B). Fagopyrins are a group of at least eight red- to violet-colored crystalline compounds with strong adhesion to glass that appear pink when dissolved in DMSO and fluoresce orange under 366 nm light [14,24]. Fagopyrins fluoresce orange to red under UV light at 366 nm. Fluorescence detection was done using an excitation wavelength of 330 nm and an emission wavelength of 590 nm [14]. So, in the present study with UV spectroscopic analysis, we monitored the presence of fagopyrins compounds in the wavelength ranging from 360.8 nm. The hypericin, protofagopyrin, fagopyrin A, fagopyrin E, and fagopyrin F are currently known, and these five yet remaining to be elucidated [12]. As spectrum 420–430.4 is typical for piperidine [25], we suggested that the identified compound may contain 2-(piperidine-2-yl) (Figure 1B). The isolated compound was attached to cationite and visible separation of emodin was observed. As result of such experimental observations, we concluded that 2-(piperidine-2-yl)-emodindianthron can be a possible precursor for farther steps of fagopyrin synthesis.

**Figure 2:**
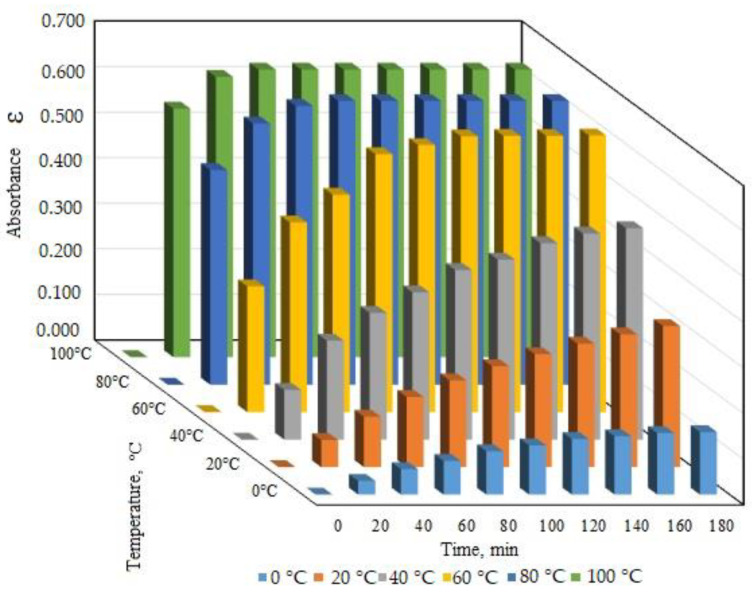
Identified absorbance spectrum of protofagopyrin under different temperature treatment conditions (90% acetone).

The protofagopyrin formation during the process of naphthodianthrone isolation in vitro was found to be possible under the influence of temperature and may depend on the duration of temperature treatment. The temperature effect on protofagopyrin formation was studied in the range from 0 to 100 °C with the identification of protofagopyrin absorbance during 180 min (or 2 h). The experimental study of protofagopyrin formation under different temperature conditions showed that the fastest protofagopyrin formation was during 20 min under the temperature 80 °C and 100 °C. Nearly 1 h was needed for protofagopyrin formation under the temperature 60 °C (Figure 2). 

Quantitively analysis and calculation of protofagopyrin content showed mostly similar levels for 80 °C and 100 °C temperature treatments. It was estimated to be 0.64 ± 0.03 mg per g DW for variants with the temperature treatment of 80 °C.

**Figure 3:**
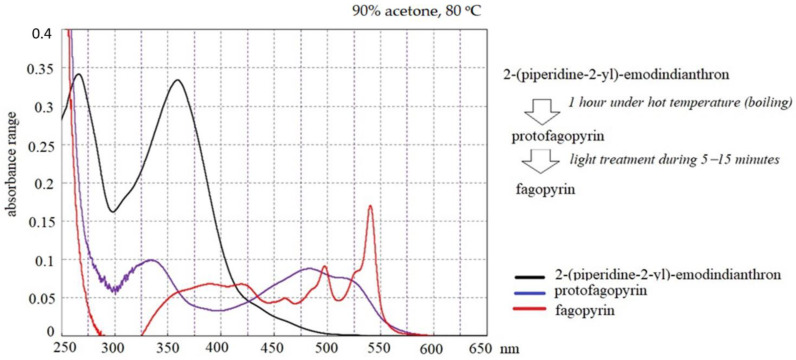
Absorbance spectrum of compounds analyzed by differential spectrophotometry.

The identified absorbance spectrum for fagopyrin and protofagopyrin were in the range from 549–593 nm (Figure 1A), which are similar to hypericin absorbance [26] but their identification were done under further extraction condition in vitro with the use of temperature treatment and light exposition (Figure 3). One of the scientific theories of fagopyrin formation is supported by the experiment where protofagopyrins were observed in a light-protected extract sample using an HPLC-DAD instrument as a weak signal at 590 nm; they showed that the retention times were similar, but not equal, to those of fagopyrins from an identical but light-exposed sample. Before light exposure, there were no fagopyrin peaks in the sample. In the UV−vis spectra, a strong absorbance maximum at 590 nm was observed upon exposure to light, indicating the formation of fagopyrins [14]. The present study found that fagopyrin formation in vitro can be influenced by high temperature in case of formation of protofagopyrin from 2-(piperidine-2-yl)-emodindianthron and by light effect during 5–15 min for fagopyrin formation.

The content of 2-(piperidine-2-yl)-emodindianthron, protofagopyrin, and fagopyrin determined in the different steps by differential spectrophotometry is presented in Table 2. It is visible that content of studied 2-(piperidine-2-yl)-emodindianthron, protofagopyrin and fagopyrin are in the range from 0.67 mg per g DW to 0.70 mg per g DW.

## 3. Discussion

Kim and Hwang, 2020 identified the presence of fagopyrin A–E using mass-spectrometry analysis with a major peak for fagopyrin F in the chromatogram in samples from the different parts of common and tartary buckwheat during their flowering period [13]. The presence of different forms of fagopyrins was confirmed but a detailed analysis of their characterisitics and their biosynthesis was not done. The results of the present study with the use of differential spectroscopy showed in intact plants the likely presence of 2-(piperidine-2-yl)-emodindianthron (precursor of fagopyrin) with further formation of protofagopyrin and fagopyrin under conditions in vitro. The spectrum range for the identification of fagopyrin precursor (piperidine-2-yl)-emodindianthron was ranging from 360.8 nm to 430.4 nm. Milan et al., 2013 found that spectrum in the range 420–430.4 is typical for piperidine, which helped with the conclusion that the identified compound can be 2-(piperidine-2-yl) [25]. At the same time, the isolated compound was attached to cationite with further visible separation of emodin, which showed the presence of emodin. Huang et al., 2014 described more than 9 known schemes in total synthesis, semi-synthesis, and biosynthesis of hypericin. Emodinanthrone condensation can be done via oxidation to protohypericin in pyridine solution in the presence of a little piperidine in a stream of air [27]. In 1999, Falk et al. reported a simple semi-synthetic method, which begin with emodin. Emodin was decreased by stannous chloride to produce emodinanthrone, which was then treated with a solution of pyridine and ferrous sulfate to obtain the condensation product hypericin [28].

Emodin is a precursor in the synthesis of hypericin and fagopyrin [29]. Emodin is a chemical compound of the anthraquinone family, which is characterized by antiviral, antibacterial, and anti-inflammatory effects, among others [30]. It has also been identified as having a potential antiviral activity against coronaviruses such as SARS-CoV-2 [31,32]. In our previous studies, fagopyrin and hypericin were shown to have antifungal and antibacterial properties as well [10,11].

It was observed that fagopyrin formation in vitro can be influenced by high temperature during the phase of protofagopyrin formation and by light effect during 5–15 min for fagopyrin formation. Quinones, like fagopyrin, and also hypericin, express a light-dependent activity [33].

At the same time, the present experimental study of buckwheat 90% aqueous acetone extracts under different temperature conditions showed that the fastest protofagopyrin formation during 20 min under the temperature 80 °C and 100 °C. Nearly 1 h is needed for protofagopyrin formation under the temperature 60 °C. Toleikytė et al., 2016, used 80% aqueous acetone as the extracting agent for nathtodianthrones extraction from buckwheat (*Fagopyrum* sp.) and ST. John’s wort (*Hypericum perforatum* L.) herbs. The extraction time was set as 10 min at 65 °C. Extraction was repeated one more time for a complete extraction of naphthodianthrones [21]. Temperature was found to influence the extraction process of fagopyrin compound, which was confirmed with a more detailed analysis and its connection with time duration in our study.

**Figure 4:**
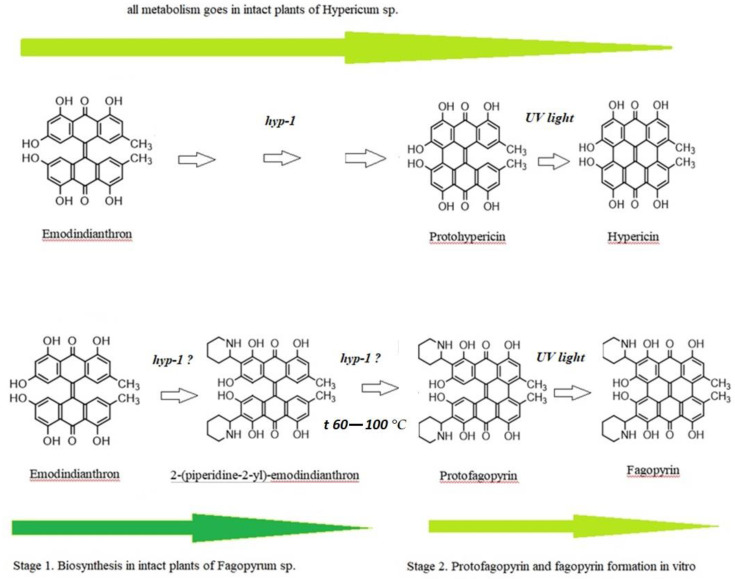
The suggested fagopyrin and hypericin biosynthesis with updates based on the presented experimental results.

Differential spectroscopy is an elegant and powerful analytical method, based on the relatively simple principles of classical absorption spectroscopy, which can be implemented during pharmaceutical development, in production for process monitoring, and in quality control laboratories [34]. The use of differential spectroscopy over an extended wavelength range has several major advantages, which we also observed in our experimental study with fagopyrin derivatives’ estimation. In the studied range 360–430 nm, mostly 2-(piperidine-2-yl)-emodindianthron was estimated in the buckwheat extracts. The estimated absorbance spectrum for fagopyrin and protofagopyrin were in the range from 549–593 nm with the combined analysis of temperature treatment (boiling) and light effect (LED lamp with a power of 10 watts). The protofagopyrin content was identified with the temperatures 80 °C and 100 °C for a time duration of 20 min. The light effect for 15 min was possible to identify fagopyrin content. Quantitative analysis of 2-(piperidine-2-yl)-emodindianthron, protofagopyrin, and fagopyrin determined in the different steps by differential spectrophotometry showed their content to be in the range from 0.67 mg per g DW to 0.70 mg per g DW. Ozbolt et al. (2008) determined in the leaves of common buckwheat (*Fagopyrum esculentum* L.) fagopyrin content in a range 0.44–0.64 mg per g DW [16]. The experimental results of differential spectrophotometry analysis of green part of buckwheat may show missing gaps in the fagopyrin biosynthesis, which previously were known as emodinanthrone-protofagopyrin-fagopyrin biosynthesis [26]. The suggested fagopyrin and hypericin biosynthesis with updates based on the presented experimental results are presented in Figure 4.

Conversion of the protoforms to hypericin and pseudohypericin goes under the effect of light, but protein enzyme hyp-1 has also been suggested to participate in these reactions [35,36,37]. The chemical synthesis of hypericin has been achieved by following the pattern of the biosynthesis, using emodidianthron as a precursor [38,39]. In vivo, hypericin is synthesized via emodin dimerization in a complicated multistep reaction. This reaction is catalyzed by a small (17.8 kDa) protein, Hyp-1 [37]. We suppose that emodidianthron is a precursor for 2-(piperidine-2-yl)-emodindianthron and protofagopyrin. There is a possibility that fagopyrin synthesis is a multistep reaction that may be catalyzed by protein Hyp-1 or a similar one. The 2-(piperidine-2-yl)-emodindianthron was found to be in high quantity in buckwheat plants. We also suppose that emodidianthron is a precursor for 2-(piperidine-2-yl)-emodindianthron and protofagopyrin. The last reaction of fagopyrin synthesis is protofagopyrin conversion to fagopyrin on exposure to light [40]. In HPLC methods which were developed by Eguschi et al., 2009, vials were exposed to a light for 24 h at 4 °C to convert protofagopyrin to fagopyrin [17]. Alali et al., 2004, have reported that protohypericin in hypericin-containing HPLC vials is converted to HYP on exposure to light immediately before HPLC analysis [41], which shows a difference in hypericin and fagopyrin formation. The light influence was confirmed by the current study as well. Sha et al.,2019, standardized the optimum extraction conditions for fagopyrins analysis as follows:an extraction time of 1.5 h, 85% acetone, and an extraction temperature of 55 °C [19]. Toleikytė et al., 2016, standardized another optimum extraction condition for fagopyrins analysis but showed the same connection with temperature. The extraction was done under 65 °C. [21]. At the same time Hinneburg and Neubert, observed that an extract with a high content of phenolics and antioxidant activity but with a low content of the phototoxic fagopyrin can be obtained by agitated maceration with 30% ethanol at 60 °C for 2 h [20]. As we see, temperature factor is very important for the optimal and faster extraction process and is confirmed by the present study as well. It was found that nearly 1 h was needed for the protofagopyrin formation under the temperature of 60 °C. Under temperature 80–100 °C, protofagopyrin formation was observed during 20 min. As fagopyrin can be produced by light effect, the temperature factor may influence the formation of protofagopyrin in vitro.

## 4. Material and Methods

### 4.1. Plant Material

The buckwheat (*Fagopyrum esculentum* L.) plants were grown in the experimental field of Department of Plant Biology, Educational and Scientific Center “Institute of Biology and Medicine”, Taras Shevchenko National University of Kyiv, Ukraine. The plant material for fagopyrin and hypericin determination was collected at the flowering period from *Fagopyrum esculentum* L. Cultivar Rubra of *Fagopyrum esculentum* L. with high anthocyanins content (3.87–4.41 mg/100 g DW) in the vegetative organ has been received by family selection method from chemo mutants from Taras Shevchenko National University of Kyiv [2]. The vegetative part was collected and frozen in liquid nitrogen to prevent the volatilization of phenolics. Later, samples were lyophilized.

### 4.2. Quantitative Determination of 2-(piperidine-2-yl)-emodindianthron, Protofagopyrin and Fagopyrin by Differential Spectrophotometry

Fagopyrin was determined using the method of differential spectrophotometry developed by us. The description of this method can be found below. The exact weight of the dry material (25–50 mg) was milled in a porcelain mortar with 200 mg of glass powder. The milled substrate was transferred in a centrifuge tube. Ten milliliter of 90% aqueous acetone was added to it, fixed with cap, shaked, and placed in a thermostat at different temperatures (20 °C, 40 °C, 60 °C, 80 °C, 100 °C) for 2 h. The optimal temperature for the analysis of 2-(piperidine-2-yl)-emodindianthron and protofagopyrin was found to be 80 °C. The reaction mixture was shaken vigorously for the first 15 min.

In a further step for the identification of 2-(piperidine-2-yl)-emodindianthron from 2 experimental test tubes, 4 mL of extract was taken from each and transferred to centrifuge tubes. To one aliquot 50 mg of Dowex 50 × 8 (200–400 mesh) cationite in H^+^ form was added and mixed for 5 min. This was followed by centrifugation at 2000× *g* for 3 min.

After the incubation in a thermostat, the tube with the experimental mixture was irradiated with a sunlight stream for 15 min. An LED lamp with a power of 10 watts 10W (GLS LED Light Bulbs B22 BC Bayonet Paul Russells, Dublin, Ireland) was used and kept at a distance of 10–20 cm from the glass test tube containing the mixture. As a result of rapid illumination, the mixture in the test tube started precipitating, and so the extract was centrifuged at 2000× *g* for 3 min.

The absorbance of the mixture for protofagoyrin and fagopyrin was evaluated at 593 nm. The absorbance for 2-(piperidine-2-yl)-emodindianthron was evaluated at 430.4 nm. An aliquot-treated cation extract was used as an optical control. The experimental samples were measured spectrophotometrically with spectrophotometer UV-1800 (Shimadzu, Tokyo, Japan). The quantity of fagopyrin is determined as follows:

(1) $$x=\frac{D\times MW\times V}{\varepsilon \times a}$$

where, *x* is the protofagoyrin, fagopyrin content in the sample, mg∙g^−1^; *D* is the optical density of extract; *MW* is the molecular weight of fagopyrin, 670.71; *V* is the volume of extract, ml; *ε* is the coefficient of molar extinction of hypericin, 43,700; *a* is the weight of plant material, g.

### 4.3. Statistical Analysis 

Complete findings are represented as mean ± SE, and alterations between means were analyzed using the ANOVA program, providing statistical significance (*p* < 0.05) for each result.

## 5. Conclusions

The developed method of differential spectrophotometry can be used for the identification of naphthodianthrones fagopyrins. The precursor of fagopyrin in its native form 2-(piperidine-2-yl)-emodindianthron was identified via the method of differential spectrophotometry as a major compound in intact buckwheat plants. It was observed that temperature factor may influence formation of protofagopyrin in vitro. It was shown that nearly 1 h was needed for the formation of protofagopyrin at a temperature of 60 °C. Under temperature 80–100 °C, protofagopyrin formation was observed during 20 min. The suggested fagopyrin and hypericin biosynthesis scheme with updates based on the presented experimental results showed possible difference in metabolism in the intact plants of *Fagopyrum* sp. and *Hypericum* sp. The developed method of differential spectrometry analysis and results of this study can be used in further studies. It is recommended to use described method together with mass spectrometry analysis to analyze the presence of fagopyrins in the different buckwheat cultivars and species.

## Figures and Tables

**Table 1:** The known parameters of extraction of fagopyrin for spectrophotometric analysis.

Method	Plant Weight	Solvent for Extraction	Time and Conditions of Extraction	Supernatant Solvent	Wavelength	References
A spectrophotometric method	80 mg	80% tetrahydrofuran	80% tetrahydrofuran in water at 65 °C for 30 min	methanol	590 nm	[16]
A spectrophotometric method	5 g	30% ethanol	freeze drying for 24 h at −30 °C	methanol	590 nm	[20]
A spectrophotometric method	-	80% acetone	10 min at 65 °C.	Solid-Phase extraction with column, methanol	590 nm	[21]
A spectrophotometric method	105 mg	90% acetone extract under temperature 55 °C	1.5 h at 55 °C	90% acetone	590 nm	[19]

**Table 2:** The content of 2-(piperidine-2-yl)-emodindianthron, protofagopyrin, and fagopyrin determined by differential spectrophotometry.

Compound	Conditions of Determination by Differential Spectrophotometry	Content mg per g DW
2-(piperidine-2-yl)-emodindianthron	Extract with 90% aquatic acetone	0.64 + 0.05
protofagopyrin	80 °C temperature treatment, duration 20 min	0.64 + 0.03
protofagopyrin	60 °C temperature treatment, duration 60 min	0.70 + 0.04
fagopyrin	LED lamp with a power of 10 watts, 10–15 min	0.69 + 0.07

## Data Availability

Not applicable.

## Sample Availability

Samples of the compounds are not available from the authors.
